# Isolation and whole-genome sequencing of *Pseudomonas* sp. RIT 623, a slow-growing bacterium endowed with antibiotic properties

**DOI:** 10.1186/s13104-020-05216-w

**Published:** 2020-08-03

**Authors:** KayLee K. Steiner, Anutthaman Parthasarathy, Narayan H. Wong, Nicole T. Cavanaugh, Jonathan Chu, André O. Hudson

**Affiliations:** Thomas H. Gosnell School of Life Sciences, Rochester Institute of Technology, 85 Lomb Memorial Drive, Rochester, NY 14623 USA

**Keywords:** Pseudomonas, Slow-growing, Whole-genome sequencing, Aquatic, Antibiotics, Drug discovery

## Abstract

**Objective:**

There is an urgent need for the discovery and/or development of novel antibiotics. We report an exploration of “slow”-growing bacteria, which can be difficult to isolate using rich media as they are usually outcompeted by “fast”-growing bacteria, as potential sources of novel antimicrobials.

**Results:**

*Pseudomonas* sp. RIT 623 was isolated using pond water agar from a pond located on the campus of the Rochester Institute of Technology (RIT). The genome was sequenced and analyzed for potential secondary metabolite gene clusters. Bioinformatics analysis revealed 14 putative gene clusters predicted to encode pathways for the anabolism of secondary metabolites. Ethyl acetate extracts from spent growth medium of *Pseudomonas* sp. RIT 623 were tested against two Gram-negative (*E. coli* ATCC 25922 and *P. aeruginosa* ATCC 27853) and two Gram-positive (*B. subtilis* BGSC 168 and *S. aureus* ATCC 25923) type strains to assess antibiotic activity. The antibiotic assays demonstrated that extracts of *Pseudomonas* sp. RIT 623 were able to inhibit the growth of the four strains. The active compound was separated using diethyl ether in a multi-solvent extraction and reverse phase chromatography. The bioactive compound/s were subsequently eluted in two consecutive fractions corresponding to approximately 16–22% acetonitrile, indicative of polar compound/s.

## Introduction

The treatment of bacterial infections changed with the introduction of antibiotics. Lack of stewardship, coupled with evolution and additional factors led to bacteria becoming resistant to clinically relevant antibiotics. Antibiotic research declined for a number of reasons including the fact that medicines for lifestyle diseases are more profitable [1, 2]. However, there is a revival in research to combat antibiotic resistant bacteria [3]. Novel antibiotics are urgently needed especially since the rate of resistance is typically faster than the rate of discovery [4].

Apart from the Actinomycetes [5], marine antibiotic producers have been exploited [6–8], but freshwater isolates remain underexplored in bioprospecting [9]. The diversity of antibiotic producing isolates has been limited by the use ofrich media inherently biased towards the selection of “fast-growing” bacteria. Strategies have been designed to isolate slow-growing bacteria, especially Actinobacteria, with a view to exploit their antimicrobial properties [10–12]. A simple method to isolate slow-growing bacteria based on modified agar media is known [13]. Similarly, “pond agar” this study enabled the isolation of a “slow”-growing *Pseudomonas* sp. from a pond located on the campus of the Rochester Institute of Technology (RIT), which produces unidentified broad-spectrum antibiotics active against *Escherichia coli*, *Pseudomonas aeruginosa*, *Bacillus subtilis* and *Staphylococcus aureus*.

## Main text

### Methods

#### *Isolation of* Pseudomonas *sp. RIT 623*

Water samples were obtained from a pond on the RIT campus, and vacuum-filtered through a 0.22 μm sterile Corning filter. Filtered pond water and agar were mixed to make a 1.5% minimal media “pond agar”. The plates were inoculated with unfiltered pond water and incubated at 30 °C for 28 days. Pure colonies were isolated from pond agar, subsequently streaked on Tryptic Soy Agar (TSA), Luria Broth agar (LB) and Reasoner’s 2A agar (R2A), and grown at 30 °C for 24 h.

#### Growth experiments for doubling time determination

One hundred milliliters of LB medium was inoculated with a colony of RIT 623 and incubated at 30 °C with shaking at 120 rpm. Samples were withdrawn from 0–52 h and the absorbance at 600 nm was recorded on a Thermo Scientific Genesys 20 spectrophotometer, blanking with un-inoculated media. The doubling times was determined using the online Doubling time calculator Version 3.1.0 [14].

#### Genomic DNA isolation

Genomic DNA was extracted from 1.5 mL of the bacterium grown in Tryptic Soy Broth at 30 °C for 24 h. The GenElute Bacterial Genomic DNA Kit (Sigma-Aldrich, St. Louis, MO) was used according to the manufacturer’s instructions and quantified using a Nanodrop One spectrophotometer.

#### Whole-genome sequencing and assembly

Using the Nextera XT library prep kit, genomic DNA was processed following manufacturer’s protocol (Illumina, San Diego, CA). The library was normalized to a concentration of 4 nM in molecular-grade water. Of the resulting 4 nM library, 5 µL was then pooled with other tagmented, indexed, and normalized libraries. The pooled libraries were denatured and diluted to a loading concentration of 12 pM. The pooled, denatured and diluted 12 pM library was sequenced using the MiSeq Reagent Kit V3 on the Illumina MiSeq for 2 × 151 cycles at the RIT Genomics Facility. Adapter trimming was performed automatically on the Illumina MiSeq during FASTQ generation. Trimmed reads were uploaded to the Galaxy web platform, and assembled de novo at the public server at usegalaxy.org [15] using Unicycler version 0.4.6.0 [16], with a minimum contig length of 200 bp.

#### Predictions of secondary metabolite production

The assembled genome sequence of *Pseudomonas* sp. RIT 623 was analyzed using the Antibiotics and Secondary Metabolite Analysis Shell (antiSMASH) 5.0 webserver [17].

#### Phylogenetic analysis

The assembled FASTA contig file for *Pseudomonas* sp. RIT 623 was uploaded to the Type Strain Genome Server (TYGS) on 2019.09.26 and taxonomically classified [18]. The ten closest strains in the TYGS database to the query assembly were determined using Mash, a whole-genome clustering method [19]. Another ten closest relatives to the query assembly were selected by Basic Local Alignment Search Tool (BLAST) [20]. 16S rDNA sequences extracted from the query using RNAmmer [21] against all 10342 type strains in the TYGS database, selecting the top 50 hits, and calculating the Genome BLAST Distance Phylogeny distance (GBDP) with the query genome to determine the closest 10 type strains [22].

Pairwise distances between genomes (the FASTA assembly query, 10 closest BLAST hits and 10 closest GBDP hits) were calculated using GBDP and intergenomic distances were calculated under the algorithm “trimming”, using distance formula d_5_. Based on Genome–Genome Distance Calculator guidelines [22], Digital DNA–DNA Hybridization values and confidence intervals were calculated. Inter-genomic distances were used to infer a balanced minimum evolution tree using FASTME 2.1.4 [23]. Branch support was inferred from 100 pseudo-bootstrap replicates each, and the resulting trees were visualized with PhyD3 [24]. Additionally, a delta statistic was calculated for the GBDP tree based on the initial distance matrix of GBDP values. A delta statistic assesses phylogenetic accuracy in terms of tree-likeness; lower delta statistic values imply higher phylogenetic accuracy [25].

#### Scanning electron microscopy

Scanning Electron Microscopy (SEM) samples were prepared following an open source protocol [26], and covered with gold–palladium for 2 min with an SPI sputter coater to mitigate charging in the electron beam. SEM was performed at 5 kV using a Mira3Tescan field emission instrument at the RIT Nano-Imaging Lab.

#### Extraction of organic compounds from culture media

*Pseudomonas* sp. RIT 623 was grown in a starter culture of 100 mL LB broth at 30 °C for 24 h shaking at 130 rpm which was used to inoculate 900 mL of LB broth and grown for 48 h. Following centrifugation, the spent media was acidified to pH 2.0, and saturated with sodium chloride. A liquid–liquid extraction with ethyl acetate was repeated three times, the organic phases were pooled and dried over anhydrous Na_2_SO_4_. The solvent was evaporated on a Cole-Parmer SB-1300 rotary evaporator, the residue dissolved in 100% methanol, divided into 1 mL aliquots, dried using a speed-vac (Savant) with a refrigerated condenser trap and stored at −20 °C.

#### Compound fractionation and liquid chromatography

Instead of a single-solvent extraction as described in Sect. “Extraction of organic compounds from culture media”, spent media was sequentially extracted with hexanes, toluene, diethyl ether, dichloromethane and ethyl acetate. The concentrated diethyl ether sample was subjected to semi-preparative scale liquid chromatography using a Zorbax Eclipse XDB-C18 reverse phase column (9.4 × 250 mm; 5 μm, Agilent Technologies) on a Biorad NGC-10 system (Biorad, Hercules, CA) equipped with a BioFrac fraction collector. The mobile phase consisted of vacuum-filtered and degassed 0.1% aqueous formic acid (buffer A) and HPLC grade acetonitrile (Buffer B). The chromatography was performed at flow rate = 3 mL/min, fraction size = 3 mL, and detection wavelength = 280 nm, in the following order: 10% B (2 min), gradient 10–95% B (30 min), 95% B (3 min), gradient 95–10% B (7 min) and 10% B (5 min). Pooled fractions were concentrated and stored as described in Sect. “Extraction of Organic Compounds from Culture Media”.

#### Antibiotic tests

*E. coli* ATCC 25922, *P. aeruginosa* ATCC 27853, *B. subtilis* BGSC 168 and *S. aureus* ATCC 25923 were grown overnight in LB medium at 30 °C with shaking at 130 rpm. These were re-suspended in sterile phosphate buffered saline (PBS) and mixed with warm LB agar in a petri dish. Six millimeter sterile blank paper disks (BD Biosciences, USA) were aseptically placed onto the agar. Methanol, tetracycline stock (10 mg/mL), and the extracts (ethyl acetate, compound fractionation, and liquid chromatography) were pipetted onto the disks and the plates were incubated for 16 h at 30 °C. Zones of inhibition were measured based on the clearance of the bacterial lawn. The disk assays were replicated three times.

### Results

#### Strain characterization and phylogeny

Pond agar simulates the natural growth environment of the bacterium, thus de-selecting “fast”-growing strains, enabling the growth of “slow”-growing aquatic strains. Out of the seven strains, RIT 623 was chosen since it had the most number of potential antibiotic biosynthetic gene clusters (BGC) (Table 1 and Additional file 1: Table S1). *Pseudomonas* sp. RIT 623 grows on standard laboratory media, in addition to the pond agar that it was isolated on. The doubling time of our strain in LB broth was 7.4 h, as calculated by an online doubling time calculator [14]. In laboratory media, “fast”-growing *E. coli, P. aeruginosa* and *S. aureus* have doubling times of 0.33, 0.5 and 0.4 h, respectively [27]. Pathogenic *Pseudomonas* strains have “medium” doubling times of approximately 2–3 h [28]. “Slow”-growing organisms may be characterized by doubling times longer than 2–3 h. Based on SEM images (Additional file 2: Figure S1), *Pseudomonas* sp. RIT 623 is approximately 2.5 μm × 0.75 μm, with multiple flagella and projections on the periphery of the cell. The flagella could contribute to biofilm formation since flagella and twitching motility are necessary for biofilm development in the hyper-virulent *P. aeruginosa* PA14 [29].

**Table 1:** Summary of antiSMASH 5.0 results for *Pseudomonas* sp. RIT 623

Region	Type of metabolite	From	To	Most similar known cluster	Similarity
2	NAGGN	42,676	57,575	–	–
6	NRPS-like	33,465	75,141	–	–
*6*	*PpyS-KS*	*186,487*	*197,805*	*Pseudopyronine A/pseudopyronine B*	*43%*
15	NRPS	66,508	107,419	Pyoverdine	11%
27	Bacteriocin	48,531	59,358		
*37*	*NRPS*	*1*	*37,589*	*Sessilin*	*55%*
47	NRPS	1	30,407	Pyoverdine	6%
*60*	*NRPS-like*	*1*	*15,414*	*Mangotoxin*	*71%*
62	NRPS	1	14,591	Pyoverdine	9%
*63*	*NRPS*	*1*	*14,095*	*Putisolvin*	*62%*
64	NRPS	1	13,527	Pyoverdine	3%
*68*	*NRPS*	*1*	*9118*	*Rhizomide A/rhizomide B/rhizomide C*	*100%*
*71*	*NRPS*	*1*	*8037*	*Rhizomide A/rhizomide B/rhizomide C*	*100%*
*81*	*NRPS-like*	*1*	*1419*	*Xenotetrapeptide*	*100%*

The analysis detected 81 regions of the genome as potential metabolic gene clusters, out of which only 13 contain gene clusters related to antibiotics. The numbering of the nucleotides corresponds to the location of each gene cluster in that particular region (and does not correspond to the coordinates published for the whole genome sequence). The gene clusters with  ≥ 50% similarity to known biosynthetic gene clusters are shown in bold

*NAGGN* N-γ-acetylglutaminyl glutamine 1-amide, *NRPS* non-ribosomal peptide synthases, *PpyS-KS* PPY-like pyrone cluster

Initial 16S V3/V4 rRNA and subsequent whole-genome sequencing using the BLAST identified RIT 623 as a *Pseudomonas* sp [30]. After sequencing and assembly, the genome sequence was submitted to the National Center for Biotechnology Information (NCBI), under the accession number SOZA00000000. The Prokaryotic Genome Annotation Pipeline (PGAP) predicted 4814 protein-coding sequences, 3 rRNAs, and 67 tRNAs. An assembly of the genomic sequence generated 98 contigs with a total length of 5400,139 bp, an N_50_ value of 108,051 bp, and a GC content of 64.41%. *Pseudomonas* sp. RIT 623 was placed into taxonomic context using TYGS. The resulting tree (Additional file 3: Figure S2) had an average branch support of 95.1% and a delta statistic of 0.199. *Pseudomonas* sp. RIT 623 had digital DNA–DNA hybridization (dDDH) values of 66.9% and 60.4% with *Pseudomonas soli* LMG 27941 and *Pseudomonas entomophila* L48, respectively. Both values fall below the accepted boundary for dDDH-based species delimitation of 70%, indicating that *Pseudomonas* sp. RIT 623 is a novel species. In terms of GC content, *Pseudomonas* sp. RIT 623 differs from *P. soli* LMG 27941 and *P. entomophila* L48 by 0.94% and 1.1%, respectively.

#### Secondary metabolism and antibiotic activity

Genome mining by antiSMASH 5.0 resulted in the prediction of 14 gene clusters in 13 regions associated with secondary metabolite biosynthesis (Table 1). Many of the BGC are predicted to encode non-ribosomal peptide synthetases (NRPS) (Table 1), which produce antibiotics [31]. High similarity to BGC in databases were also present for BGC encoding xenotetrapeptide (100%; structurally uncharacterized [32]), the rhizomide A/B/C family (100%; isolated from Burkholderia spp. [33]), mangotoxin (71%; *P. syringae* NRP with unknown structure [34], targeting arginine/ornithine biosynthesis [35]), putisolvin (62%; *P. putida* cyclic lipopeptide [36]), sessilin (55%; *Pseudomonas* sp. lipopeptide [37]) and pseudopyronine A/B (43%; produced by *P. putida* BW11M1 [38]). Among BGC with potential biosynthetic novelty (low similarity) are four putative BGC encoding pyoverdines (Table 1). Pyoverdines are well-known fluorescent siderophores with roles in regulating pathogenicity and iron metabolism [39]. Pyoverdine analogs competitively inhibit pyoverdine uptake in *P. aeruginosa* [40]. A putative BGC for bacteriocin is found, whose product may inhibit the growth of closely related species [41].

**Fig. 1:**
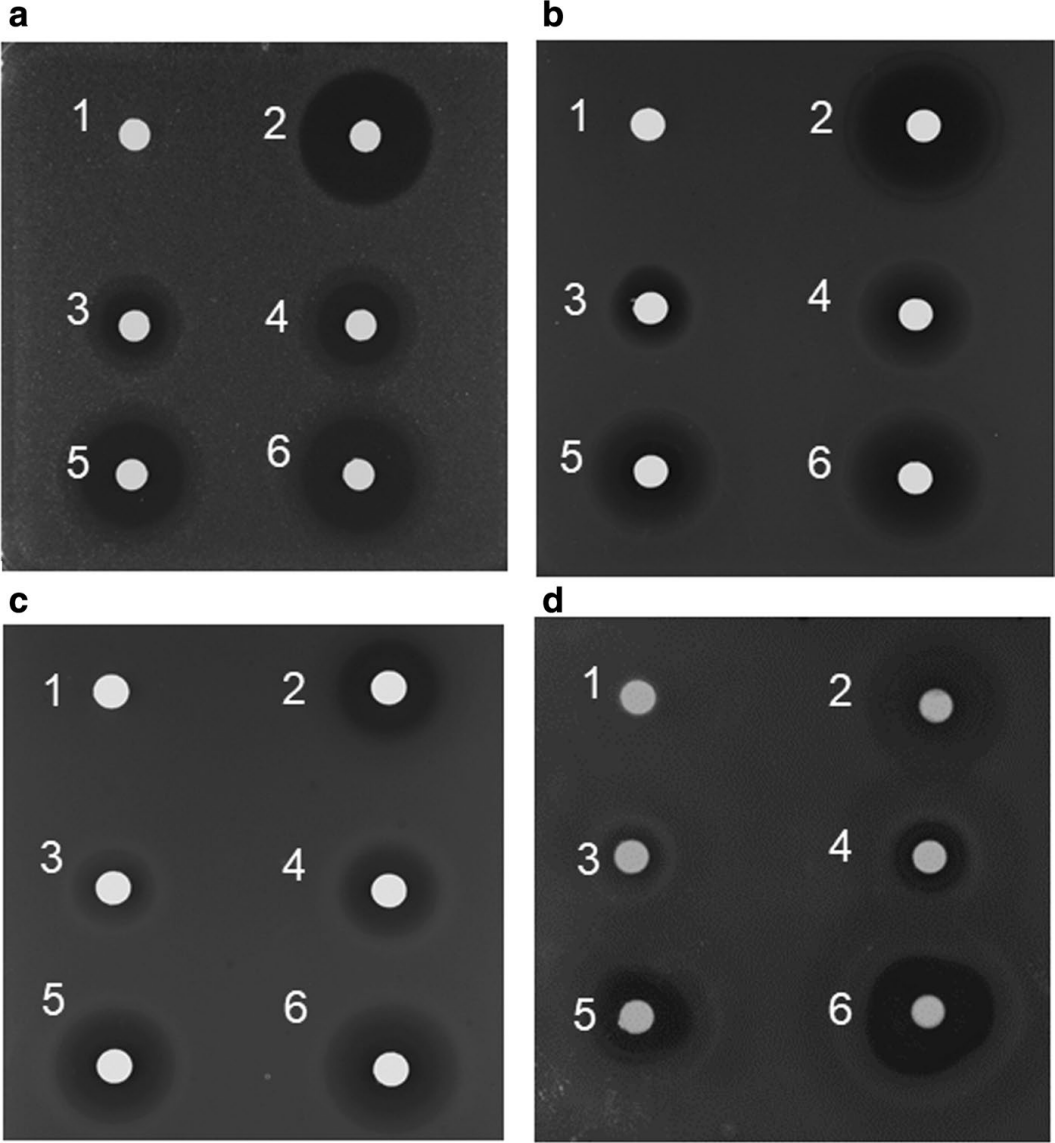
**a-d** Disk diffusion assay using ethyl acetate extract of *Pseudomonas* sp. RIT 623. Against **a***Bacillus subtilis* BGSC 168, **b***Staphylococcus aureus* ATCC 25923. **c***Escherichia coli* ATCC 25922, **d***Pseudomonas aeruginosa* ATCC 27853. (1) Methanol, 20 μL; (2) Tetracycline, 20 μL (10 mg/mL); and (3) 10 μL, (4) 20 μL, (5) 40 μL, and (6) 60 μL of *Pseudomonas* sp. RIT 623 extract dissolved in 100% methanol, respectively

**Fig. 2:**
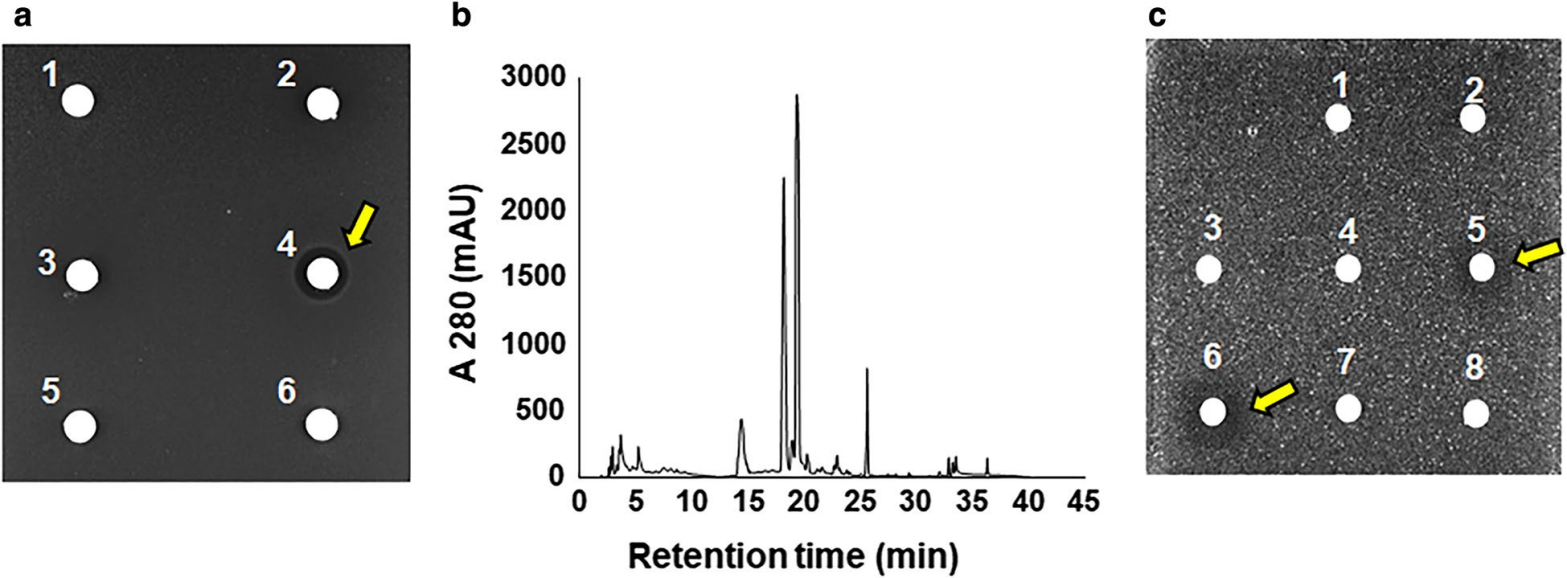
Separation of compounds using liquid extractions and liquid chromatography. **a** Disk diffusion assay using five extracts from spent medium of RIT 623 against *Bacillus subtilis* BGSC 168. From the top left (50 µl each), (1) Methanol (negative control) (2) Hexanes (3) Toluene (4) Diethyl ether (5) Dichloromethane (6) Ethyl acetate. Only the diethyl ether is active as seen in the clear inhibition zone (yellow arrow). **b** Chromatogram of ether extract from the five step extraction (refer to methods) of RIT 623 spent medium using a reverse phase chromatography on a C18 semi-preparative scale column. The elution profile is based on the collection of a 3 mL fraction every minute at a flow rate of 3 mL/min. **c** The activity of fractions 1–8 pooled and concentrated from four runs of the chromatography experiment in **b**) are shown. The active compound eluted in fractions 5 and 6 (yellow arrows). There was no activity in the pooled fractions 8–42 (data not shown)

For in vivo studies, positive correlations of the diameter of the zones of inhibition using extracts from *Pseudomonas* sp. RIT 623 against *B. subtilis*, *S. aureus, E. coli,* and *P. aeruginosa* were observed (Fig. 1; Additional file 4: Table S2). These bacteria are representative non-pathogenic type strains related to known pathogens for screening of antibiotic activity, without involving Biosafety level 2/3 procedures. Only the diethyl ether extract was active among those extracts tested (Fig. 2a). Further separation by liquid chromatography (Fig. 2b), led to the elution of an unknown polar antibacterial compound at 16–22% acetonitrile (Fig. 2c).

### Discussion

*Pseudomonas* sp. produce antibiotics, such as the topical medication, mupirocin [42, 43]. Genome mining of the plant pathogens, *P. corrugate* and *P. mediterranea*, revealed the presence of predicted BGC for siderophores, polyketides, NRPs and hydrogen cyanide [44]. Aquatic antibiotic producers have recently been isolated, including P*. donghuensis* from a lake that makes plant-probiotic, antibacterial, antifungal and siderophore compounds [45–47], and aquatic gliding bacteria from the genera *Flavobacterium* and *Lysobacter* whose metabolites inhibit several pathogens [9]. Slow-growing RIT 623 possibly produces antibiotics to inhibit fast-growing species in a nutrient-limited environment, and slow-growing freshwater bacteria in general may yield novel antibiotics.

## Limitations

The chemical structure/s of the bioactive compound/s that are endowed with antibiotic activities are still unknown and will form the basis for future experiments aimed at identifying and elucidating the sturctures.

## Supplementary information

**Additional file 1: Table S1.** Predicted biosynthetic gene clusters (BGC) of the other isolates on pond agar. The data for strain 623 is shown in Table 1 of the main article.

**Additional file 2: Figure S1.** Scanning electron microscopy of *Pseudomonas* sp. RIT 623. The magnifications are × 37640 (**a**), × 65420 (**b**) and × 88600 (**c**). White arrows in (**a**) and (**b**) indicate the fine filamentous flagella, while in (**c**), they point to the ridged/wavy appearance of the cell borders. Cells are joined together by bridging projections along the periphery.

**Additional file 3: Figure S2.** A Genome Blast Distance Phylogeny (GBDP) tree. Pairwise genomic distances were calculated between RIT623 and its ten closest relatives, as determined by 16S rDNA gene sequence similarity. Resulting inter-genomic distances were used to generate a minimum evolution tree with branch support via FASTME 2.1.4. Branch support, indicated at each node, was inferred from 100 pseudo-bootstrap replicates.

**Additional file 4: Table S2.** Comparison of zones of inhibition (ZOI) against various reference strains using ethyl acetate extracts. Average values over three replicates are reported and the standard deviation is indicated in parenthesis.

## Data Availability

The whole-genome project for Pseudomonas sp. RIT 623 is available in GenBank with the accession number SOZA00000000 (BioProject number PRJNA528499; BioSample number SAMN11192858).

## Abbreviation

RIT: Rochester Institute of Technology
TSA: Tryptic soy agar
LB: Luria Broth
R2A: Reasoner’s 2A
antiSMASH: Antibiotics and secondary metabolite analysis shell
TYGS: Type strain genome server
BLAST: Basic local alignment search tool
GBDP: Genome BLAST distance phylogeny
SEM: Scanning electron microscopy
PBS: Phosphate buffered saline
NCBI: National Center for Biotechnology Information
PGAP: The prokaryotic genome annotation pipeline
dDDH: Digital DNA–DNA hybridization
BGC: Biosynthetic gene clusters
NRPS: Non-ribosomal peptide synthases
