# Supplementary material for: Isolation and whole-genome sequencing of Pseudomonas sp. RIT 623, a slow-growing bacterium endowed with antibiotic properties
Source: BMC Res Notes. 2020 Aug 3;13:370. doi: 10.1186/s13104-020-05216-w (PMC7398229; doi:10.1186/s13104-020-05216-w)
Supplement: Supplementary file 1 — Additional file 1: Table S1. Predicted biosynthetic gene clusters (BGC) of the other isolates on pond agar. The data for strain 623 is shown in Table 1 of the main article. [file 13104_2020_5216_MOESM1_ESM.docx]

**Supplementary Table 1. Predicted biosynthetic gene clusters (BGC) of the other isolates on pond agar.** The data for strain 623 is shown in Table 1 of the main article.

| Strain no. | Predicted biosynthetic metabolite | Coordinates within the genome | % similarity to known cluster |
| --- | --- | --- | --- |
| 624 | Arylpolyene | 202506-246110 | 40% APE Vf, Other |
|  | Nrpsfragment | 525168-557004 | 71% Mangotoxin, NRPS |
|  | Bacteriocin | 193819-204703 |  |
|  | Nrps | 6225-59223 | 21% Pyoverdine, NPRS |
|  | Belalactone | 71732-94984 | 13% Fengycin, hybrid |
|  | Bacteriocin | 40465-51352 |  |
|  | Nrps | 1-39267 | 10% Pyoverdine, NRPS |
| 625 | Nrps | 6225-59223 | 21% Pyoverdine, NRPS |
|  | Belalactone | 67480-90732 | 13% Fengycin, hybrid |
|  | Nrpsfragment | 228424-260259 | 71% Mangotoxin, NRPS |
|  | Bacteriocin | 54220-65104 |  |
|  | Nrps | 1-44,443 | 10% Pyoverdine, NRPS |
|  | Arylpolyene | 20903-64507 | APE Vf, other |
|  | Bacteriocin | 40456-51343 |  |
| 626 | Terpene | 65328-86173 |  |
|  | Hserlactone | 102138-122794 |  |
|  | Ectoine | 64734-75132 | 66% Ectoine, other |
| 627 | Nrps | 6225-59223 | 21% Pyoverdine, NRPS |
|  | Nrpsfragment | 1-31836 | 71% Mangotoxin, NRPS |
|  | Arylpolyene | 310894-354498 | 40% APE Vf, other |
|  | Baceriocin | 94696-105580 |  |
|  | Belalactone | 71732-94984 | 13% Fengycin, hybrid |
|  | Nrps | 1-44463 | 10% Pyoverdine, NRPS |
|  | Bacteriocin | 40470-51357 |  |
| 629 | Terpene | 598584-619429 |  |
|  | Hserlactone | 101224-121880 |  |
|  | Ectoine | 290200-300598 | 66% Ectoine, other |
| 630 | Bacteriocin | 661414-672241 |  |
|  | T1pks-Otherks | 34917-87686 |  |
|  | Arylpolyene-Ladderane | 98324-142113 | 25% Xenocyloins, polyketide |
|  | Resorcinol-Arypolyene | 194416-227114 |  |
|  | Nrps | 16544-72814 | 22% Taiwachelin, NRPS |
|  | Bacteriocin | 100720-111562 |  |
|  | Belalactone | 191504-208554 | 20% Fengycin, hybrid |
|  | Arylpolyene | 23148-66747 | 40% APE Vf, other |

**Supplementary Table 2. Comparison of zones of inhibition (ZOI) against various reference strains using ethyl acetate extracts.** Average values over three replicates are reported and the standard deviation is indicated in parenthesis.

| Volume of extract (μL) | Average ZOI (mm) | | | |
| --- | --- | --- | --- | --- |
|  | *P. aeruginosa ATCC 27853* | *S. aureus*  *ATCC 25923* | *E. coli*  *ATCC 25922* | *B. subtilis*  *BGSC 168* |
| 10 | 10.3 (4.9) | 10.6 (2.9) | 12.3 (2.5) | 12.3 (4.7) |
| 20 | 12.6 (6.4) | 14.6 (6.4) | 15.3 (2.3) | 15.6 (5.1) |
| 40 | 15.3 (6.6) | 19.3 (6.6) | 20.3 (2.5) | 20.0 (4.3) |
| 60 | 17.6 (7.2) | 21.6 (7.5) | 23.6 (1.5) | 22.6 (4.2) |
