# Supplementary figures and images for: Isolation and whole-genome sequencing of Pseudomonas sp. RIT 623, a slow-growing bacterium endowed with antibiotic properties
Source: BMC Res Notes. 2020 Aug 3;13:370. doi: 10.1186/s13104-020-05216-w (PMC7398229; doi:10.1186/s13104-020-05216-w)

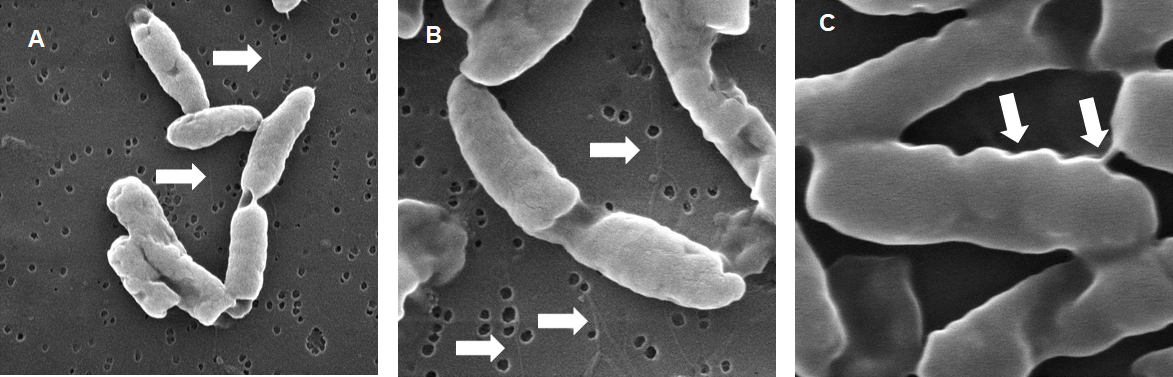

Supplement: Supplementary file 2 — Additional file 2: Figure S1. Scanning electron microscopy of Pseudomonas sp. RIT 623. The magnifications are × 37640 (a), × 65420 (b) and × 88600 (c). White arrows in (a) and (b) indicate the fine filamentous flagella, while in (c), they point to the ridged/wavy appearance of the cell borders. Cells are joined together by bridging projections along the periphery. [file 13104_2020_5216_MOESM2_ESM.tif]

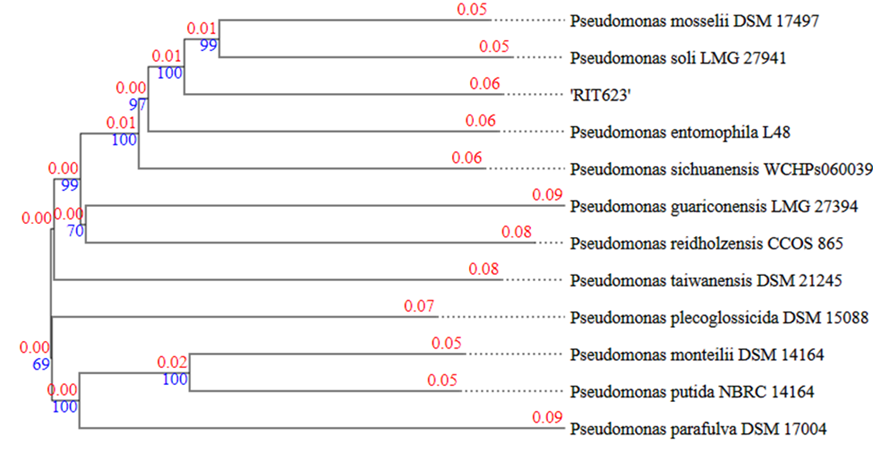

Supplement: Supplementary file 3 — Additional file 3: Figure S2. A Genome Blast Distance Phylogeny (GBDP) tree. Pairwise genomic distances were calculated between RIT623 and its ten closest relatives, as determined by 16S rDNA gene sequence similarity. Resulting inter-genomic distances were used to generate a minimum evolution tree with branch support via FASTME 2.1.4. Branch support, indicated at each node, was inferred from 100 pseudo-bootstrap replicates. [file 13104_2020_5216_MOESM3_ESM.tif]
