# Supplementary material for: Isolation and whole-genome sequencing of Pseudomonas sp. RIT 623, a slow-growing bacterium endowed with antibiotic properties
Source: BMC Res Notes. 2020 Aug 3;13:370. doi: 10.1186/s13104-020-05216-w (PMC7398229; doi:10.1186/s13104-020-05216-w)
Supplement: Supplementary file 4 — Additional file 4: Table S2. Comparison of zones of inhibition (ZOI) against various reference strains using ethyl acetate extracts. Average values over three replicates are reported and the standard deviation is indicated in parenthesis. [file 13104_2020_5216_MOESM4_ESM.docx]

**Additional file 4: Table S2. Comparison of zones of inhibition (ZOI) against various reference strains using ethyl acetate extracts.** Average values over three replicates are reported and the standard deviation is indicated in parenthesis.

| Volume of extract (μL) | Average ZOI (mm) | | | |
| --- | --- | --- | --- | --- |
|  | *P. aeruginosa ATCC 27853* | *S. aureus*  *ATCC 25923* | *E. coli*  *ATCC 25922* | *B. subtilis*  *BGSC 168* |
| 10 | 10.3 (4.9) | 10.6 (2.9) | 12.3 (2.5) | 12.3 (4.7) |
| 20 | 12.6 (6.4) | 14.6 (6.4) | 15.3 (2.3) | 15.6 (5.1) |
| 40 | 15.3 (6.6) | 19.3 (6.6) | 20.3 (2.5) | 20.0 (4.3) |
| 60 | 17.6 (7.2) | 21.6 (7.5) | 23.6 (1.5) | 22.6 (4.2) |
